# Effects of Antidiabetic Drugs on Gut Microbiota Composition

**DOI:** 10.3390/genes8100250

**Published:** 2017-09-30

**Authors:** Sophie A. Montandon, François R. Jornayvaz

**Affiliations:** Service of Endocrinology, Diabetes, Hypertension and Nutrition, Geneva University Hospitals, Rue Gabrielle-Perret-Gentil 4, 1205 Geneva, Switzerland; sophie.montandon@unige.ch

**Keywords:** gut microbiota, type 2 diabetes, antidiabetic drugs, metformin, incretins

## Abstract

Gut microbiota forms a catalog of about 1000 bacterial species; which mainly belong to the Firmicutes and Bacteroidetes phyla. Microbial genes are essential for key metabolic processes; such as the biosynthesis of short-chain fatty acids (SCFA); amino acids; bile acids or vitamins. It is becoming clear that gut microbiota is playing a prevalent role in pathologies such as metabolic syndrome; type 2 diabetes (T2D); inflammatory and bowel diseases. Obesity and related diseases; notably type 2 diabetes, induce gut dysbiosis. In this review; we aim to cover the current knowledge about the effects of antidiabetic drugs on gut microbiota diversity and composition as well as the potential beneficial effects mediated by specific taxa. Metformin is the first-line treatment against T2D. In addition to its glucose-lowering and insulin sensitizing effects, metformin promotes SCFA-producing and mucin-degrading bacteria. Other antidiabetic drugs discussed in this review show positive effects on dysbiosis; but without any consensus specifically regarding the Firmicutes to Bacteroidetes ratio. Thus, beneficial effects might be mediated by specific taxa.

## 1. Introduction

The gut microbiota refers to the trillions of microbes living in our guts and can be considered a separate endocrine organ [1]. The composition and richness of the gut microbiota depends on the symbiotic relationship with the host. They are modulated by the diet, host health, age, ethnicity and genetics and thus are unique and highly variable among individuals [2,3]. This variability renders comparison between studies difficult. Therefore, there are some discrepancies among studies. Nevertheless, Turnbaugh and colleagues [4] suggest that there is a “core gut microbiome” (i.e., shared microbial genes) that could be necessary for proper gut functioning. The gut microbes are predominantly bacteria, which belong to the Gram-positive Firmicutes and the Gram-negative Bacteroidetes. The gut is also colonized by other phyla such as Actinobacteria, Proteobacteria and Verrucomicrobia [5,6]. Intestinal bacteria provide amino acids and vitamins to its host. They influence bile acid pool size and composition, for example by generating unconjugated and secondary bile acids, and as such are essential for bile acid homeostasis [7,8,9]. Moreover, one main function of gut microbiota is to breakdown non-digestible carbohydrates into short-chain fatty acids (SCFA), mostly acetate, propionate and butyrate. Consequently, gut microbiota contributes to a broader metabolic potential for the host [5]. Albeit, the side effect of gut microbiota is the induction of low-grade inflammation, mainly through the innate immune system and the pattern recognition receptors (PRRs) [10]. Because of the interactions between gut microbiota and host physiology, it is becoming evident that the gut and its associated bacteria play a prevalent role in diseases and especially in metabolic syndrome [6,11,12]. The metabolic syndrome combines multiple disorders including insulin resistance, dyslipidemia, hyperglycemia, hypertension and central obesity [13]. It is becoming a leading cause of mortality and morbidity in industrialized countries because it increases the risk of developing type 2 diabetes (T2D) or cardiovascular diseases [12,14,15]. In this review, we will focus on gut dysbiosis associated to obesity and T2D and the effects of currently used non-insulin antidiabetic drugs on gut microbiota. Initially, we will briefly discuss gut dysbiosis associated with obesity and T2D and we will report rodent and human studies that assess the role of biguanides, α-glucosidase inhibitors, incretin-based drugs and other less studied medications on gut microbiota (summarized in Table 1). Finally, we will discuss possible origins of discrepancies among studies and the role of SCFA on human physiology.

## 2. The Effects of Antidiabetic Treatments on Gut Microbiota Composition

The relationship between diseases such as metabolic syndrome, obesity or T2D and gut microbiota composition has been extensively reviewed elsewhere (ex: [6,30,31,32,33,34,35]). Briefly, even though there are still some discrepancies among studies, it has been shown that both energy-rich diets and obesity tend to increase the intestinal Firmicutes to Bacteroidetes ratio in humans and murine models [6,36,37,38,39]. Obesity also contributes to a reduction of microbial diversity [31]. Likewise, T2D induces a dysbiosis, characterized by a decrease in the butyrate-producing bacteria abundance [19,30] and in *Akkermansia muciniphila*, which was further proposed to be a biomarker for glucose intolerance [40]. Using the observed dysbiosis associated with T2D, Karlsson et al. [41] developed a model based on gut metagenome profiles to diagnose T2D in human patients, but also to identify individuals with a high risk of developing T2D. They further showed that these metagenomic predictive tools need to be population-specific.

### 2.1. Change in Lifestyle

Probably the most efficient therapy against obesity and related diseases is a modification of lifestyle, namely diminution of high-energy food intake and inactivity. Gut microbiota composition and diversity is predominantly influenced by the diet [6,34,42]. It has been shown in mice that dietary changes can explain 57% of variation in gut microbiota structure vs. only 12% for genetic mutation [43]. Moreover, energy-rich diets can alter gut microbiota composition in a single day [39]. High-fat diet promotes taxa belonging to the Firmicutes (*Clostridium ramosum*) and Proteobacteria (*Bilophila wadsworthia*), while high-fiber diet increases the abundance in Bacteroidetes (*Prevotella*, *Xylanibactera*), Verrucomicrobia (*Akkermansia muciniphila*) and Actinobacteria (*Bifidobacterium* spp.) and decreases the amount of noxious Proteobacteria (*Shigella*, *Escherichia*) [6,44]. 

On the other hand, physical activity modifies gut physiology and morphology in addition to gut microbiota composition [45]. Studies in rodents have shown that voluntary exercise leads to an increase in n-butyrate caecal concentration and that the Firmicutes to Bacteroidetes ratio decreases proportionally to the total distance run [46,47]. Furthermore, Evans and colleagues [47] showed that exercise in diet-induced obese mice is able to restore a microbial composition similar to lean mice.

### 2.2. Biguanides

Amongst biguanides, metformin is currently used as first-line treatment of T2D for its glucose lowering and insulin sensitizing effects [48,49]. Even though the multiple mechanisms of action of metformin are currently not fully understood, it is suggested to regulate glucose uptake, gluconeogenesis, glycolysis and glycogen synthesis in the liver. Moreover, metformin improves insulin-mediated glucose uptake in skeletal muscle and modulates the incretin pathway by enhancing the expression of glucagon-like peptide-1 (GLP-1) receptor in the pancreas islets and increasing plasma levels of GLP-1 [49,50]. Interestingly, intravenous administration of metformin does not improve glycaemia [51]. Metformin treatment also alters bile acid recirculation [52] suggesting that the primary actions of metformin could be in the gut [30]. Furthermore, some beneficial actions, but also side-effects of metformin are thought to be mediated by gut microbiota [19,25,52]. 

In rodents, metformin has been shown to modify gut microbiota composition and diversity, but in a diet-dependent manner [25,26]. Indeed, Shin and colleagues [25] showed that there are significant differences in gut microbiota composition and in the abundance of Firmicutes and Bacteroidetes, between metformin-treated and non-treated mice, but only under a high-fat diet (HFD). Similarly, Lee and Ko [26] observed that metformin induces a decrease in bacterial diversity in mice on a HFD. 

Metformin contributes to the enrichment of SCFA-producing bacteria such as *Blautia*, *Bacteroides*, *Butyricoccus* or *Phascolarctobacterium* and has positive effects on the Proteobacteria phylum as well as *Allobaculum* and *Lactobacillus* genera [22]. Furthermore, the relative abundance of the phylum Verrucomicrobia increases in metformin-treated mice on a HFD, mostly because of the genus *Akkermansia* [22,25,26]. This positive influence is probably due to metformin action on mucin-producing goblet cells in the intestine. The abundance of *Akkermansia*, which are mucin-degrading bacteria, is positively correlated with the number of goblet cells. Note that metformin increases the number of goblet cells independently of the diet [25].

In humans with T2D, metformin treatment modulates genes involved in amino acids degradation and enhances the potential to produce butyrate and propionate [19,20]. Similarly to rodents, *Akkermansia* and *Lactobacillus* relative abundances are increased by metformin treatment as well as SCFA-producing bacteria such as *Bifidobacterium*, *Prevotella*, *Megasphaera* and *Butyrivibrio* [19,20,21]. Moreover, metformin enriches human feces in *Escherichia* and decreases *Intestinibacter* relative abundance [19,20]. Whereas *Escherichia* probably plays an important role in the side-effects of metformin [19], *Bifidobacterium adolescentis* has been shown to negatively correlate to HbA1c. Therefore, this taxon could contribute to the glucose-lowering effect of metformin [20]. 

To summarize, in both humans and rodents, metformin acts on pathways that include mucin-degradation and SCFA-production. As suggested by Shin et al. [25], restoration of relative abundance of specific genera could play a role in the antidiabetic effects of metformin. Even though the mechanisms of action of biguanides are not clearly understood, their oral administration probably have both direct and indirect effects on gut bacteria. It has notably been shown that metformin impairs folate metabolism in *Escherichia coli* [53], possibly by the inhibition of the dihydrofolate reductase activity [54]. In vitro, metformin is able to promote the growth of *Akkermansia muciniphila* and *B. adolescentis*, which indicates that this drug may be a growth factor for some bacterial species [20,26]. However, metformin is not able to enrich *E. coli* in vitro and rather shows antibiotic effects [20,53]. Therefore, metformin action on the *Escherichia* genus could be both direct and indirect.

### 2.3. Alpha-Glucosidase Inhibitors

Alpha-glucosidase inhibitors (α-GIs) are antidiabetic drugs that delay the digestion of carbohydrates, such as disaccharides and starch, in the small intestine, and reduce postprandial hyperglycemia. Thus, α-GIs affect the nutrient sources of bacteria by partitioning complex carbohydrates. Interestingly, efficient α-GIs are of microbial origins and have been postulated to favor their producers in a community competing for the same nutrients [55]. The α-GI acarbose is able to block the maltose importer and consequently the growth of *E. coli* on maltose [56]. Given the direct and indirect effects of α-GIs on bacteria metabolism, it is not surprising that they influence gut microbiota composition. In mice, miglitol was shown to shorten the intestinal transit time as well as to suppress histological and molecular markers of inflammation induced by a high fat and high glucose diet [27]. Moreover, miglitol reverses the increase in Erysipelotrichaceae and Coriobacteriaceae generated by the energy-rich diet. These modifications in gut microbiota have been postulated to be related to the suppression of intestinal inflammation [27]. Similarly to rodents, miglitol is able to modify the human gut environment by reducing the transit time [57], but nothing is known about its effects on human gut microbiota diversity and composition.

Acarbose increases the fecal concentrations of starch and butyrate, but reduces the amount of propionate. This suggests that acarbose prevents starch processing and absorption and enhances starch-fermenting and butyrate-producing bacteria, at the same time, it inhibits starch use by propionate-producing bacteria [58]. Acarbose administration in hyperlipidemic or T2D patients was further shown to increase *Lactobacillus* and *Bifidobacterium* [23,24,29] as well as other SCFA-producing bacteria such as *Faecalibacterium* and *Prevotella* [24]. Moreover, Zhang and colleagues [24] showed that the increased abundance of *Dialister* after acarbose treatment is negatively correlated with HbA1c, which indicates a probable role for species belonging to this taxon in the regulation of glucose metabolism. Finally, acarbose treatment was also associated with a diminution of Enterobacteriaceae, Bacteroidaceae and lecithinase positive *Clostridum* in human feces [23]. 

As previously mentioned, high-fat diets increase the Firmicutes to Bacteroidetes ratio and lowers the abundance in Verrucomicrobia. Voglibose, another α-GI, reverses this dysbiosis in diet-induced obese mice [16]. The authors suggest that these favourable changes could enhance the production of bile acid metabolites and have a beneficial systemic effect. 

### 2.4. Incretin-Based Drugs

Incretins are small peptide hormones secreted by intestinal cells after meal absorption. They have pleiotropic metabolic actions and play a major role in the regulation of blood glucose levels and the reduction of appetite. As such, in the past decade they have become a major target to treat T2D and related metabolic disorders [59,60]. Currently, two peptides are recognized as incretins: the glucose-dependent insulinotropic polypeptide (GIP, or gastric inhibitory peptide) and the GLP-1. GIP and GLP-1 are secreted by the gut within minutes after food ingestion by intestinal K and L cells respectively [61]. Both incretins are mainly inactivated by the enzyme dipeptidyl peptidase-4 (DPP-4) and endogenously co-occur as an active and an inactive form [62]. In addition to enzymes, incretin metabolites are cleared by renal filtration, thus endogenous half-life of these peptides is very short [63]. In order to increase the beneficial effects of incretins, two categories of incretin-based drugs have been developed: (i) GLP-1 receptor agonists (GLP-1 RA) and (ii) DPP-4 inhibitors (DPP-4i). 

Even though this section focuses on the effects of incretin-based drugs on gut microbiota, it is worth mentioning that gut microbiota fermentation products also influence incretin secretion. Acetate and propionate are able to stimulate GLP-1 secretion in primary murine colonic cultures via the G-protein-coupled free fatty acid receptor (FFAR)2 and FFAR3 [64]. 

#### 2.4.1. GLP-1 Receptor Agonists

GLP-1 RA are modified peptides sharing homology with GLP-1 [65]. Despite the fact that direct effects of GLP-1 RA injected subcutaneously on the gut microbiota composition are not expected, Wang and colleagues [18] observed substantial rearrangement of the bacterial structure of mice treated with liraglutide. They postulated that GLP-1 levels, that influence the gut transit time and gastric emptying rate, could modify the gut lumen internal environment (local pH value and nutrient composition) and thus affect microbiota composition. In mice fed a HFD, liraglutide reduces microbial diversity. While only the Firmicutes are enriched, the Bacteroidetes, Proteobacteria and Actinobacteria phyla are depleted. Furthermore, in HF-fed or diabetes-induced mice, liraglutide treatment induces enrichment in 13 phylotypes in genera *Allobaculum*, *Turicibacter*, *Anaerostipes*, *Blautia*, *Lactobacillus*, *Butyricimonas*, *Desulfovibrio*, whereas it decreases 20 phylotypes in the orders Clostridiales and Bacteroidales [18]. Similarly to other GLP-1 RA, liraglutide is known to induce weight loss [66]. Therefore, Wang et al. [18] looked for lean-and obesity-related phylotypes influenced by liraglutide treatment. Among the seven genera and three families associated with weight decrease, liraglutide promoted only *Lactobacillus*, *Turicibacter*, *Blautia* and *Coprococcus*, while it reduced all obesity-related phylotypes (*Erysipelotrichaceae incertae sedis*, *Marvinbryantia*, *Roseburia*, *Candidatus Arthromitus* and *Parabacteroides*). Note that in this study, the DPP-4i saxagliptin was able to decrease the relative abundance of only one phylotype associated with weight gain, the genus *Candidatus Arthromitus* [18]. 

Currently, only few studies show the actions of GLP-1 RA on gut microbiota and we could not find any data on other GLP-1 RA such as exenatide, dulaglutide, albiglutide or lixisenatide.

#### 2.4.2. DPP-4 Inhibitors

Amongst DPP-4i, sitagliptin was shown to restore the gut microbiota structure at the phylum level in diabetic-induced rats, without significant modifications in body weight. Sitagliptin induces an increase in the relative abundance of Bacteroidetes and Proteobacteria and a decrease in Firmicutes. At the genus level, sitagliptin influences SCFA-producing bacteria. After treatment, diabetic rat stools are enriched in *Roseburia* and depleted in *Blautia*, while the relative abundance of *Clostridium* do not change [17]. Yan et al. [17] also showed that probiotics such as *Lactobacillus* and *Bifidobacterium* are depleted in feces from diabetic rats. However, sitagliptin was able to prevent only the *Bifidobacterium* reduction and seems to exacerbate *Lactobacillus* decrease. 

Interestingly, saxagliptin, another DPP-4i, seems to have rather opposite effects on the microbiota phyla distribution than sitagliptin, but we cannot exclude that these differences are due to the model (rat vs. mouse) rather than the drug. The feces of HF-fed or diabetic-induced mice treated with saxagliptin are enriched in Firmicutes, mainly because of the genera *Lactobacillus*, *Allobaculum* and *Turicibacter*. In addition, they contain less *Bacteroides* and *Prevotella*, which induces a decrease in the phylum Bacteroidetes [18]. 

Studies are currently lacking regarding the effect of other DPP-4i such as alogliptin, vildagliptin or linagliptin, on gut microbiota. 

### 2.5. Other Anti-Diabetic Drugs

We could not find any study showing the influence of sodium glucose co-transporter (SGLT) 2 inhibitors or meglitinides on gut microbiota and information is very scarce regarding sulfonylureas and thiazolidinediones. Sulfonylureas have been used against diabetes for more than 50 years. They promote insulin secretion by inducing β-cell depolarization (Quianz 2012). Data is lacking regarding the action of sulfonylureas on gut microbiota composition. However, Huo et al. [67] suggested that sulfonylureas have a beneficial effect on gut metabolism of T2D patients based on their urine levels of hippurate, phenylalanine and tryptophane. 

Thiazolidinediones, such as rosiglitazone and pioglitazone, are peroxisome-proliferator-activated receptor (PPAR)—γ agonists. They decrease insulin resistance in adipose tissue, liver and skeletal muscle [68]. In addition to their antidiabetic properties, thiazolidinediones can have multiples effects such as anti-tumoral, anti-inflammatory, anti-bacterial or anti-fungal [69] and may indirectly affect gut bacteria. We could find only one study by Bai et al. [28] that discusses the effects of thiazolidinediones on gut microbiota. They show that diet-induced obesity in rats is associated with an increase in inflammatory markers levels (tumor necrosis factor alpha (TNF-α), interleukin (IL)-6 and monocyte chemoattractant protein (MCP)-1) and a reduction in the anti-inflammatory cytokine IL-10. In comparison to normal chow, HFD leads to an increase in the relative abundance of Proteobacteria, which highlights the potential role for this phylum in the inflammation associated with obesity. Pioglitazone administration in HF-fed rats reduces the abundance of Proteobacteria, which seems to correlate with the decrease of plasma endotoxin, TNF-α, IL-6 and MCP-1 levels. On the other hand, pioglitazone exacerbates the drop in IL-10 level and the authors did not observe any significant decline in the endotoxin-producing families Enterobacteriaceae and Desulfovibrionaceae [28]. Thus, pioglitazone seems to only partially rescue gut dysbiosis and inflammation resulting from a HFD.

## 3. Discussion

Gut microbiota co-evolves with its host and adapts to the environment in which it resides, namely the digestive tract. Therefore, changes in lifestyle observed in “western” countries, i.e., where there is a high-energy food intake and low physical activity, lead to modifications in gut microbiota composition and diversity. This observed dysbiosis could play a role in the exacerbation of diseases such as obesity, metabolic syndrome or T2D. Thus, it is important to characterize gut dysbiosis and identify key taxa involved in such diseases. However, there is currently no clear consensus. 

In this review, we summarized the current knowledge about the effects of antidiabetic drugs on gut dysbiosis (Table 1). Not surprisingly, the most studied medication is the first-line T2D treatment metformin. Studies in rodents and humans tend to agree that metformin treatment increases the relative abundance of SCFA-producing bacteria and in particular the genera *Akkermansia*. Research articles are very scarce or even non-existent concerning the other antidiabetic drugs. Studies on DPP-4i do not reach any consensus, but rather show opposite results. However, it is difficult to know if these variations are due to the medication (sitagliptin vs. saxagliptin) or to the studied model (rats vs. mice).

It is likely that the main variation amongst studies is the model (human, mouse, rat, “humanized” mouse/rat etc.), but the strain/population will also influence the results [2,70]. These inter-specific variations question the validity of rodent studies to cure human diseases [71]. Mice and humans show similarities in their digestive tract, however, their macroscopic (ex: caecum, colon) and microscopic (ex: mucosa thickness) anatomy as well as cell distribution (ex: goblet and Paneth cells) are different, generating diverse ecological micro-niches [71]. Still, approximately 80 genera are shared between humans and mice regardless of differences in their relative abundance [70,71]. Furthermore, gut microbiota is not homogenous along the digestive tract especially between mucosa (caecum or colon) and stool [3]. Thus, analyses made on caecal or fecal samples will generate different results. The methods used to analyze gut microbiota can also generate disparities. While most studies use 16S rDNA sequencing approaches to identify bacterial operational taxonomic unit (OTUs), metagenomic sequencing is becoming popular due to a decrease in sequencing costs. Finally, the taxonomic level at which comparisons are made can also lead to inconsistencies. In particular, the Firmicutes to Bacteroidetes ratio, which provides an analysis at the phylum level, is probably too simplistic and misleading as it can hide important variations at the genus or species levels. This further highlights the difficulties to perform meaningful meta-analyses or study comparisons.

All of the above-mentioned variations as well as the inter-individual diversity, influenced by diet, host health, age, ethnicity and genetics, probably explain the absence of a clear consensus on gut dysbiosis associated with obesity and related diseases. Albeit, most studies highlight the beneficial impact of specific taxa, for example the SCFA-producing bacteria that are positively influenced by antidiabetic drugs, high-fiber diets and physical activity. Note that similarly to comparisons made at the phylum level, the SCFA-producing bacteria “label” could be misleading as these bacteria are broadly distributed and might compete with each other [72]. SCFA are mainly produced by anaerobic bacteria from undigested starches and plant cell-wall polysaccharides. These carbohydrates are mostly catabolized into acetate, propionate and butyrate, but also lactate [73,74]. While the fermentation leading to acetate can be achieved by various bacterial taxonomic groups, production pathways of propionate, butyrate and lactate are more conserved and substrate specific [74]. Propionate production is dominated by a few bacterial genera with *Akkermansia municiphila* as a key producer [75]. The production of butyrate in the human colon is mostly achieved by *Eubacterium rectale*, *Eubacterium hallii*, *Roseburia faecis* and *Faecalibacterium prausnitzii* [76]. SCFA are strong candidates for the crosstalk between bacteria and host cells [77] and provide approximately 2.2 to 10% of human energy requirements [78,79]. Butyrate is the main energy source for colonocytes, while propionate and acetate are metabolized by the liver and other peripheral tissues. They are involved in multiple processes; they regulate gene expression and can stimulate gut peptide production [76,77]. Furthermore, butyrate shows beneficial effects on colonic carcinogenesis, inflammation and oxidative stress. It was also proposed to improve colonic barrier function and promote satiety [80].

In conclusion, SCFA and their producers seem to be crucial for human health and have been shown to be positively influenced by metformin and α-glucosidase inhibitors. However, data are scarce or even absent regarding other antidiabetic medications. In particular, there is currently a lack of knowledge about the effects of incretin-based drugs and SGLT2 inhibitors on gut microbiota of diabetic humans. It would be interesting to know if these medications could indirectly favor the reduction of glycemia and weight through modifications of gut microbiota composition. 

## Figures and Tables

**Table 1:** Effects of antidiabetic drugs on relative abundance of gut bacteria taxonomic groups.

	Biguanide	α-Gi			DPP-4i		GLP-1 RA	TZD
	Metformin	Acarbose	Miglitol	Voglibose	Sitagliptin	Saxagliptin	Liraglutide	Pioglitazone
**Firmicutes to Bacteroidetes**				↓a [16]	↓a [17]	↑a [18]	↑a [18]	
**SCFA-producing bacteria**	↑h [19,20,21], a [22]	↑h [23,24]			↑↓a [17]			
**Firmicutes**					↓a [17]	↑a [18]	↓↑a [18]	
Clostridiales (order)		↑h [24]					↓a [18]	
*Anaerostipes*							↑a [18]	
*Anaerotruncus*	↓a [25]							
*Blautia*	↑a [22]↓a [25]				↓a [17]		↑a [18]	
*Butyricicoccus*	↑a [22]	↓h [24]						
*Butyrivibrio*	↑h [21]							
*Clostridium*	↑a [26]	↓h [23]						
*Faecalibacterium*		↑h [24]						
*Intestinibacter*	↓h [19,20]							
*Lactonifactor*	↓a [25]							
*Roseburia*					↑a [17]		↓a [18]	
*Ruminococcus*		↓h [24]						
Erysipelotrichaceae (family)			↓a [27]					
*Allobaculum*	↑a [22]					↑a [18]	↑a [18]	
*Turicibacter*						↑a [18]	↑a [18]	
*Dialister*		↑h [24]						
*Lactobacillus*	↑h [19], a [22]	↑h [23,24]				↑a [18]	↑a [18]	
*Megasphaera*	↑h [21]							
*Phascolarctobacterium*	↑a [22]	↓h [24]						
**Bacteroidetes**	↑a [26]				↑a [17]	↓a [18]	↓a [18]	
Bacteroidales (order)							↓a [18]	
*Butyricimonas*							↑a [18]	
*Odoribacter*	↓a [25]							
*Prevotella*	↑h [21], a [22]	↑h [24]				↓a [18]		
Bacteroidaceae (family)		↓h [23]						
*Bacteroides*						↓a [18]		
**Verrucomicrobia**	↑h [20,21], a [22,25,26]			↑a [16]				
*Akkermansia*	↑h [20,21], a [22,25,26]							
**Proteobacteria**	↑a [22]				↑a [17]		↓a [18]	↓a [28]
*Desulfovibrio*							↑a [18]	
*Escherichia*	↑h [19,20]							
*Lawsonia*	↓a [25]							
**Actinobacteria**							↓a [18]	
*Bifidobacterium*	↑h [20,21]	↑h [23,29]			↑a [17]			
Coriobacteriaceae (family)			↓a [27]					
**Enterobacteria**								
Enterobacteriaceae (family)		↓h [23]						

↑: increased relative abundance, ↓: decreased relative abundance. In bold: phylum, in italic: genus. α-Gi: alpha-glucosidase inhibitor, DPP-4i: dipeptidyl peptidase-4 inhibitor, GLP-1 RA: glucagon-like peptide-1 receptor agonist, TZD: thiazolidinedione, h = human studies, a = animal studies.
